# Supplementary material for: Program sustainability post PEPFAR direct service support in the Western Cape, South Africa
Source: PLoS One. 2021 May 24;16(5):e0251230. doi: 10.1371/journal.pone.0251230 (PMC8189145; doi:10.1371/journal.pone.0251230)
Supplement: S1 File — (DOCX) [file pone.0251230.s001.docx]

**Health Facility Interview Guide**

*For Health Facility Manager/or their representative*

**Personal Background**

1. Please tell me a little bit about yourself.

- *Prompt****:*** *Education? How long have you worked here? Previous employment*

1. What drew you to this position?

*Analysis: Leadership (Experience)*

**Health Facility Characteristics**

1. Please tell me a bit about the health facility.

- *Prompt****:*** *Services offered, size, age of facility, type of facility*

1. From 2007-2012 what were the challenges with the *(name of health facility)* (infrastructure, supply chain, IT, etc.) that may have hindered your ability to provide quality health care services?

**Western Cape *(NGO Name)* Program 2007-2012**

**Program Description**

1. Please describe the *(NGO name)* program that was operational in your facility from 2007-2012?
2. How was the program introduced into the health facility?

- *Prompt: Were you or your staff involved with the program design?*
  - - *If yes, how were you involved?*
    - *Was there input from district/local and health facility officials?*
    - *Who was involved in the program design and program plans?*

*(e.g., program beneficiaries/local government/health facility staff)*

*Analysis: Local ownership (Perceived value)*

1. What changes did you observe within the health facility after (*NGO name)* program started?

- *Prompt:* *Health outcomes, staff motivation*

*Analysis: Health worker motivation*

1. Can you tell me a little about program communication?

- *Prompt: With NGO staff and other stakeholders?*

1. Was the program aligned with health facility governance policies and structures?

If yes, please give an example of how the program was aligned with local structures?

*Analysis: Local ownership (Integration)*

**Training**

1. Did the program offer your staff training/mentoring of any kind?

If yes, what type of training/mentoring was offered?

1. Did you find the training enhanced the quality of care provided in your health facility?

*Analysis: Health worker skill set/capacity*

**Staff**

1. Did you observe a difference in your staff when the *(NGOs name)* was supporting your health facility?

- *Prompt: Motivation*

1. Did the *(NGO name)* program increase human resources to your staff establishment?

If yes, did you find the staff helped or hindered the quality of care provided by the health facility?

*Analysis: Health worker skill set/capacity*

1. Was there someone within the *(NGO name)* or health facilities who enthusiastically advocated for the program?

If yes**,** can you tell me a little bit about them?

1. In your opinion how did this person improve the program or what value did they bring?

*Analysis: Leadership (Program champion)*

**Successes/Challenges**

1. What value did the program bring to (*health facility name)*?

*Analysis: Local ownership (Perceived value)*

1. In your opinion, did your staff and/or the health facility benefit from the program?

If yes, in what way did you benefit?

*Analysis: Program resources/activities*

1. Was there anything about the (*NGO name)* program you would have changed or modified?

- *Prompt: Challenges*

1. If there were challenges or concerns regarding the program how did you address them?

*Analysis: Supportive environment*

**Post *(NGO Name)* Program**

1. What did you think would happen when the program ended?
2. Were there any unexpected outcomes which you observed after the program ended?

*Analysis: Health worker motivation*

1. Is there anything (*health facility name)* does differently that is a result of the (*NGO name)* program?

*Analysis: Program resources/activities*

**Partnerships**

1. Were you able to build partnerships or relationships with local government officials or other civil society organizations from this program that you continue to foster?

- *Prompt: Government meetings you continue to attend*
- *If yes, how do these partnerships/relationships enhance the health facility or quality of care now?*

*Analysis: Partnerships (Communication)*

**Closing Questions**

1. Do you know of other donor or government funded HIV programs or organizations who worked in *(name of health facility)* or this district?
2. Is there anything else you would like to add?
3. Would you be willing to speak with me if I have any follow up questions?

**Thank you for your time!**

**NGO Interview Guide**

*FOR NGO Program Manager*

**Personal Background**

1. Please tell me a little bit about yourself.

- *Prompt:* *Your education? How long have you worked for (NGO name)?*

1. What drew you to this position?

**NGO Background**

1. Can you tell me a bit about the (*NGO name*)?

- *Prompt****:*** *When was the organization started? Why was it started?*

**Western Cape *(NGO Name)* Program 2007-2012**

**Program Director Role**

1. Please tell me about your role within the PEPFAR program.
2. Did you have other job responsibilities aside from the PEPFAR program?

If yes, please explain.

*Analysis: Leadership (Experience)*

**Program Description**

1. From what I understand about the program, it was doing X and X.

Is this correct?

- *Prompt: Is there anything you want to add?*

1. How was the program introduced into the health facility?

- *Prompt: Was there input from district/local and health facility officials?*

*Who was involved in the program design and program plans?*

(e.g. program beneficiaries/local government/health facility staff)

*Analysis: Local ownership (Perceived value)*

*Analysis: Leadership*

1. What changes did you observe within the health facility after (*NGO name)* program started?

- *Prompt:* *Health outcomes, staff motivation*

*Analysis: Health worker motivation*

1. Can you tell me a little about program communication?

- *Prompt: With NGO staff and other stakeholders?*

1. Was the program aligned with health facility governance policies and structures?

If yes, please give an example of how the program was aligned with local structures?

*Analysis: Local ownership (Integration)*

**Training**

1. Did you find a skills gap at a health facility level?

If yes, how did you fill the gap?

- *Prompt: What type of training/mentorship was provided?*

1. Did you find the training enhanced the quality of care provided?

If yes, please explain.

*Analysis: Health worker skill set/capacity*

**Staff**

1. Was there someone within *(NGO name)* or health facilities who enthusiastically advocated for the program?

If yes**,** can you tell me a little bit about them?

- *Prompt***:** *In your opinion how did this person improve the program or what value did they bring to the program?*

*Analysis: Leadership (Program champion)*

**Successes/Challenges**

1. In your opinion what were the successes of the program?
2. What value do you think the program brought to the (*health facility name)*?

- *Prompt: How did it enhance the quality of care provided?*

*Analysis: Local ownership (Perceived value)*

1. In your opinion, was there sufficient time to reach the program goals?
2. Were there program failures or lessons learned from the program?

- *Prompt:* *Where there any unexpected program outcomes or changes in the community or health facility you observed?*

1. Was there anything about the (*NGO name)* program you would have changed or modified?

- *Prompt: Challenges*

**Post *(NGO Name)* Program**

1. Was there a plan on how to exit the health facility?

If yes, can you please explain the plan.

1. What did you think would happen when the program ended?
2. Were there any unexpected outcomes which you observed after the program ended?

*Analysis: Health worker motivation*

**Sustainability**

1. Was sustainability a priority for the program?

If yes, please explain how sustainability was defined and implemented?

*Analysis: Local ownership (Integration)*

1. In your opinion what was sustained from the program?

*PROMPT: Outcomes*, resources, infrastructure,

**Closing Questions**

1. Do you know of other donor or government funded HIV programs or organizations who worked in *(name of health facility)* or districts?
2. Is there anything else you would like to add?
3. Would you be willing to speak with me if I have any follow up questions?

**Thank you for your time!**

**Government Interview Guide**

**Personal Background**

1. Please tell me a little bit about yourself.

- *Prompt:* *Your education? How long have you worked for local government?*

1. What drew you to this position?

**PEPFAR in the Western Cape**

1. Please tell me about how you were involved with the PEFPAR program in the Western Cape?
2. What was the PEPFAR program good at in the Western Cape?

- *Prompt: Successes*

1. What changes did you observe in the province after large PEPFAR investments were introduced?
2. Was there anything about the PEPFAR program you would have changed or modified?

- *Prompt: Challenges*

1. How did the PEPFAR and PEFPAR partners work or interact with you in your role as the HAST director?

*Analysis: Local ownership (Perceived value)*

1. Would you have changed anything with regard to their interactions with you or your unit?

*Analysis: Partnerships (Communication)*

1. In your opinion did PEFPAR leave a gap in the health system when, HIV treatment funds diminished?
2. How did the PEFPAR program enhance the Western Cape HIV program?

**Enabling Environment**

1. Can you tell me about how the Western Cape HIV policies have changed between 2007-2017?
2. What do you believe were the main challenges with regard to HIV between 2007-2012 in the Western Cape?

*Analysis: Supportive Environment*

**Closing Questions**

1. Is there anything else you would like to add?
2. Would you be willing to speak with me if I have any follow up questions?

**Thank you for your time**
