# Supplementary material for: Program sustainability post PEPFAR direct service support in the Western Cape, South Africa
Source: PLoS One. 2021 May 24;16(5):e0251230. doi: 10.1371/journal.pone.0251230 (PMC8189145; doi:10.1371/journal.pone.0251230)
Supplement: Attachment — Submitted filename: PLOS-One_Post-Pepfar Services_Review.docx [file pone.0251230.s002.docx]

PLOS-One_Review

Program Sustainability Post PEPFAR Direct Service Support in the Western Cape, South Africa

December 2020

This article provides a timely and important inquiry into the sustainability of donor-supported global health programs. This is an important topic that should have far greater attention paid to it in the global health literature. There are several aspects of the paper that require further development, including more extensive engagement with the global health literature, greater attention to the context of the Western Cape province, and relative to their proposed structure of program development, consideration of the role that community-based HIV/AIDS activists have played in South African HIV/AIDS politics and policy across the institutional levels of the state.

Thus, while the paper provides an interesting perspective on an important topic, it requires major revision before it can be considered for publication. As such, my recommendation for this paper is for it be revised and resubmitted for review.

First and foremost, there is insufficient engagement with influential approaches to program design in the field of global health. While these approaches have been primarily framed around patient-centered health outcomes rather than sustainability, since the authors focus on retention in care (RIC) as the mechanism through which sustainability is defined, the analysis is focused on similar parameters. With respect to approaches that the authors should review and engage with, Partners in Health (PiH) have advocated for an accompaniment approach to program development that involves extended engagement with across governmental sectors, civil society, and communities. In addition, Health Alliance International (HAI) has advocated for ‘diagonal’ approaches to global donor funding that involve coordination and engagement with local governmental and civil society actors and organizations to increase the impact of donor aid on communities and produce sustainable interventions. These are but two of many approaches to sustainability for global health programs that the authors would do well to consider as part of their analysis. The analysis is also quite shallow relative to the history of global/international health, which is a bit troubling. In short, the authors are not contextualizing their findings relative to important developments/histories in the field, which limits the impact and significance of their findings.

In addition, there is very little attention paid to the contextualization of this case study. I find the research to be valuable, but there is very little attention to precisely how and why the Western Cape stands out relative to other regions in South Africa relative to HIV/AIDS treatment. I have commented on this at length below and will refer the authors to my input there, but this point also links to their recommendations for program design, particularly their focus on facility managers. Taking individual leadership as a key factor without contextualizing the conditions within which facility managers operate diminishes the potential contribution. My own experience with facility managers in the Western Cape was that those who work closely with the community and HIV/AIDS activists also found success, but these sort of ties and shared governance did not find their place in the analysis or the program design recommendations the authors provide.

Regarding the parameters for the analysis, I appreciate that the authors have made the decision to publish their quantitative data separately. However, to exclude the financial data on sustainability from this paper entirely is quite limiting. This sort of data would provide the necessary context for understanding why some clinics were able to undertake the transition better than others, which remains unclear in the paper. It would also strengthen the comparative value of the paper, as we don’t know the size of Pepfar programs relative to public health budgets in the Western Cape, which would seem critical to contextualizing why the province was able to absorb former Pepfar-funded NGO staff members into the public health system. It may be the case that other societies that are Pepfar recipients would be able to undertake similar measures, but we don’t have the necessary information to undertake these sort of comparative exercises.

While it is likely beyond the scope of this paper, I would encourage the authors to think more critically about the broader power dynamics within which their research is situated. In thinking about “transitions” away from global donor funding, it is not only Pepfar that is noteworthy, but also the Global Fund. In short, this is a very important topic that they are engaging with, the implications of which are very much a life and death matter for many around the world, even more so as the COVID-19 pandemic brings with it economic contraction.

Since many recipient countries remain caught in a situation where conditionalities associated with debt repayment mean that they cannot increase health spending without commensurate increases in GDP, the looming crisis of “transitions” in global health funding mean that many societies will be facing declining levels of donor support along with shrinking health budgets. While a drug-resistant HIV epidemic is one possible entailment of this, generalized increases in mortality, under-nutrition, and suffering are also likely.

All of which is to say, I know that I have been quite critical with my review, but I have done so because I see the potential impact of this line of research and would like for the paper to reach its full potential. As such, please take my comments and critique in the constructive spirit in which they have been given, as I believe that this is an important topic that needs far greater attention.

**Comments**

Line 62

Retention in Care (RIC) – define acronym with first use

Line 105 – 108

It is true that many countries began to augment transnational donor funding during this time, but it is also important to note that the 2008 financial crisis produced a levelling-off in donor funding, and that with access to HIV/AIDS treatment and small decreases in HIV incidence, that the number of PLHIV continued to increase during this time, necessitating that recipient governments augment their HIV/AIDS programs.

Line 108-109

It would also be important to note that the World Bank recategorized country income levels according to new criteria during this time, which led to restrictions on donor flows, such as with the delineation of middle-income countries (MICs). That these criteria are based on aggregate income levels and do not take into account levels of social inequality has been an important critique of this process.

Line 140

A bit more background on why the donor community defines sustainability along financial lines is a very important issue to contextualize. From the roots of early international health programs led by the Pan-American Health Organization (PAHO), which were funded by the Rockefeller Foundation, to the rise of selective primary healthcare and cost-recovery amid structural adjustment in the 1980s, there is a clear thread whereby donors and countries that are able to exert authority within international institutions express power by defining program sustainability and impact according to criteria that they set, one of which is financial sustainability.

Line 150 – 152

Please see James Pfeiffer’s research on Mozambique on how Pepfar-funded interventions interact with public health systems, as it is more complex than these programs “working inside” public health clinics, day hospitals, etc.

Line 163-164

This is a good point, but it is also important to note that the emphasis on access to treatment was also based on the logic of treatment as prevention (TasP), or that people on HIV/AIDS treatment with undetectable viral loads could not transmit the virus to other people.

Line 169

On the lack of formal analysis of the transition in Pepfar programmatic leadership: who was to funded this? Who should have dedicated staff to examine this? There is an implicit critique here but it is left undefined, leading the reader to assume that the South African government should have done so. Is this the authors’ position? If so, please define.

Line 174

A bit more context here on why the Western Cape was distinct is important. The concentration of tertiary services and expertise is significant, but that is informed by the uneven historical development of health services around white, urban populations that began during the colonial era, continued during apartheid, and has not been resolved during the post-apartheid era. Also, HIV/AIDS programs were developed earlier in the province under the guidance of Fareed Abdullah and his team, particularly with PMTCT, which national government intervened to stop during the dark days of the Mbeki era. But support from the Global Fund in the province, which came earlier than the rest of the country, set up the Western Cape as an early success story and a province that has continued to exhibit stronger relative program management than others. Also, I believe that a similar process had already been undertaken with a Global Fund grant in the late 2000s, so there would have been institutional knowledge on how to manage the transition of donor programs from the government to an NGO.

Line 194

I hope that the financial aspects of the transition are not left out entirely, as that would weaken the robustness of the analysis considerably.

Line 200

In terms of defining RIC, a bit more would be helpful. I’m assuming that you are referring to people living with HIV/AIDS (PLHIV) who were adhering to treatment that were lost to follow up (LTFU)/did not adhere after the transition on Pepfar programs? Clarifying this would be helpful.

Line 216

Define primary healthcare (PHC) for first use

Line 232

Of what level were the staff nurses? Clinic Nurse Practitioners would be the assumption, but please define that for the reader.

Line 253

Please define modified grounded theory

Line 274

Defining the donor as an NGO is confusing here, since Pepfar is a donor program funded by the US gov’t. If you are referring to a primary recipient organization (PRO) that is working with secondary recipient organization (SRO), then that needs to be defined. Also, Pepfar, the Global Fund, and several other major global health programs are public-private partnerships (PPPs), so thinking through how you define these relationships is important, as simply labeling the donor and NGO limits the applicability and impact of your findings. Also, is the grantee always local government? This section needs to be thought through much more.

Line 277

Were there any parameters for defining what the coordinating position would be enabled to do/oversee? This seems a bit general and undefined since it is the key point in the section.

Line 280

Donor funded organization? Seems like there is a missing word here

Line 286 - 288

This is an important point that you are making, but it is not sufficiently contextualized. What you are observing is that the responsibility for a successful transition in skill transfer is decentralized and falls to the level of a facility manager. It would lead one to assume that the better capacitated facilities would therefore be better suited to have successful transfers. Since the areas with highest HIV prevalence and greatest need for skills transfer (the peri-urban townships) also tend to have over-burdened heal facilities, this is a critical point that should be further contextualized.

Line 303

Define NIMART for the reader

Line 314 – 31

Again, this had already been done by the WCDoH previously, with the transition of clinic management for the HIV/TB clinics established by Médecins sans Frontières (MSF) in Khayelitsha. There were some bumps in the road with these transitions, and it was a learning process. So, again, the success here is not surprising but the result of previous experience in navigating precisely this sort of transition.

Line 331

Again, the fact that the extra labor associated with ensuring a sustainable transition is being transferred to the facility level is really an important point. It is not being managed by the donor, provincial health, or city health, but by the facility manager. There would be a huge range of outcomes to be expected then, which would depend not only on the personal attributes of the facility manager, but the extent to which the facility is fully staffed, operational, etc. so that the manager has the ability to focus on the transition. Also, I would assume that CNPs would play a critical role in facilitating this transition, as they often have the clearest understanding of staffing needs, shortages, and areas where increased efficiencies can be achieved. Their labor, however, is often rendered invisible in this process, which is problematic.

Line 352

Define IMCI

Line 391

It is helpful that you are addressing the contextual factors here, but this is insufficient to frame your findings, which are quite particular to the Western Cape.

Line 396

It would be helpful to mention the role of HIV/AIDS activism in producing this change, as this was critical to enabling the shifts you identify

Line 400

There is a long-standing debate on the limits of vertical, disease-specific interventions and their lack of sustainability. Proponents of using “vertical” interventions to strengthen the broader health system (horizontal) interventions have advocated for doing precisely what you advise here, to create “diagonal” programs that use vertical funding streams for health systems strengthening. Health Alliance International (HAI) has done work that has modeled this approach in Mozambique is one of the most significant examples of the potential impact and success of this approach in the global health. In general, I would recommend linking your case study and discussion of findings to the global health literature, as your case study is constructed as a stand-alone example, when in reality it is part of a broader conversation on how best to channel donor funding to improve health outcomes.

Line 411

Is it because facilities are smaller or that they may be struggling to meet the level of need in communities with high burdens of disease?

Line 422

Does it make sense to mention the 90-90-90 goals for the first time in the conclusion? If this is the aim of the paper, then it would make sense to introduce this goal (which we are projected to miss significantly by the way) earlier in the paper.

Line 437

Who are national stakeholders? Does they include civil society and PLHIV or HIV/AIDS activists?

Also, shouldn’t provincial government work with facilities and communities to understand local needs?

Line 452

What are local champions? Who defines needs? What is the role of the community in this process?

Line 461

It might be useful to include the provincial treasury in the key stakeholders meetings, since presumably they will need to plan several years in advance if a transition will create greater budgetary demands for the health sector. The medium term expenditure framework (MTEF) requires such advance planning for budgetary processes.

Line 465

I would include the community or some proxy thereof in the final box in this section. The role of HIV/AIDS activists as counsellors and mentors who were also consulted by the WCDoH early on in the development of HIV/AIDS programs was vital to their success.

Line 471

Who is funding the staffing/capacity required to develop the transition plan? Is this being done by external consultants?

Line 472

The recommendation to have the skills transfer managed at the provincial level is contradicted by your evidence, which showed that facility managers oversaw this process.

Line 474

Given that the entire focus of your paper is to emphasize the importance and lack of research on how care is affected by a donor transition, I am very surprised that there is not a post-transition phase for research or monitoring/evaluation. Your proposed flow of program transition would therefore reproduce the precise issue that your paper aims to rectify.
