# Supplementary material for: Program sustainability post PEPFAR direct service support in the Western Cape, South Africa
Source: PLoS One. 2021 May 24;16(5):e0251230. doi: 10.1371/journal.pone.0251230 (PMC8189145; doi:10.1371/journal.pone.0251230)
Supplement: Attachment — Submitted filename: Responses to Reviewers .docx [file pone.0251230.s003.docx]

Dear Plos One,

Please find my responses to the reviewers below.

I would also like to thank Professor Powers for a thorough and detailed review of my work. I really appreciate the time you spent reviewing it.

Thank you.

Jessica Chiliza

1. Please ensure that your manuscript meets PLOS ONE's style requirements, including those for file naming. The PLOS ONE style templates can be found at

<https://journals.plos.org/plosone/s/file?id=wjVg/PLOSOne_formatting_sample_main_body.pdf> and

<https://journals.plos.org/plosone/s/file?id=ba62/PLOSOne_formatting_sample_title_authors_affiliations.pdf>

2. Please include additional information regarding the survey or questionnaire used in the study and ensure that you have provided sufficient details that others could replicate the analyses. For instance, if you developed a questionnaire as part of this study and it is not under a copyright more restrictive than CC-BY, please include a copy, in both the original language and English, as Supporting Information, or include a citation if it has been published previously.

3. In the Methods, please discuss whether and how the questionnaire was validated and/or pre-tested. If these did not occur, please provide the rationale for not doing so.

**I did not develop a questionnaire, but I validated the interview guide with two Health Facility Managers. I noted this on lines 294-295. I also have attached the three different interview guides I used with government leaders, NGO managers and health facility staff.**

**_______________________________________­­­­­­­­­­­­­­­­­­­_______________________________**

4. Please ensure that you refer to Figure 1 in your text as, if accepted, production will need this reference to link the reader to the figure.

**Noted and updated. Thank you.**

**_______________________________________­­­­­­­­­­­­­­­­­­­_______________________________**

5. We note you have included a table to which you do not refer in the text of your manuscript. Please ensure that you refer to Table 1 in your text; if accepted, production will need this reference to link the reader to the Table.

**Noted and edited. Thank you.**

**_______________________________________­­­­­­­­­­­­­­­­­­­_______________________________**

**Comments to the Author**

1. Is the manuscript technically sound, and do the data support the conclusions?

The manuscript must describe a technically sound piece of scientific research with data that supports the conclusions. Experiments must have been conducted rigorously, with appropriate controls, replication, and sample sizes. The conclusions must be drawn appropriately based on the data presented.

Reviewer #1: Yes

Reviewer #2: Partly

2. Has the statistical analysis been performed appropriately and rigorously?

Reviewer #1: Yes

Reviewer #2: N/A

3. Have the authors made all data underlying the findings in their manuscript fully available?

The [PLOS Data policy](http://www.plosone.org/static/policies.action#sharing) requires authors to make all data underlying the findings described in their manuscript fully available without restriction, with rare exception (please refer to the Data Availability Statement in the manuscript PDF file). The data should be provided as part of the manuscript or its supporting information, or deposited to a public repository. For example, in addition to summary statistics, the data points behind means, medians and variance measures should be available. If there are restrictions on publicly sharing data—e.g. participant privacy or use of data from a third party—those must be specified.

Reviewer #1: Yes

Reviewer #2: Yes

4. Is the manuscript presented in an intelligible fashion and written in standard English?

PLOS ONE does not copyedit accepted manuscripts, so the language in submitted articles must be clear, correct, and unambiguous. Any typographical or grammatical errors should be corrected at revision, so please note any specific errors here.

Reviewer #1: Yes

Reviewer #2: Yes

5. Review Comments to the Author

Please use the space provided to explain your answers to the questions above. You may also include additional comments for the author, including concerns about dual publication, research ethics, or publication ethics. (Please upload your review as an attachment if it exceeds 20,000 characters)

Reviewer #1: The manuscript is well written and the data and findings are presented in a clear fashion. My primary concerns with the submission is the time period in which the data is drawn. While the comparison RIC data is for the period immediately after transition, the data is five years old. There have been significant changes in the PEPFAR program since the time in which the study is conducted. At a minimum the authors should be using more recent data. Also, RIC is but one indicator which could and should be used to assess sustainability. Was there consideration given to other clinical indicators associated with the cascade, particularly HST data and viral load/suppression data?

**At the time of this study (2018-2019) the most recent RIC data at a facility level available from the Western Cape Department of Health (WCDoH) was used. While other indicators of facility performance were considered the availability and comprehensive nature of the WCDoH data lent itself to characterizing facility performance. Clinical data is not easily available at a central source that allows facility specific aggregation.**

**The indicators used to measure sustainability needed to be outcomes of the PEPFAR program. Initially we considered using 4 indicators (proportion of new HIV cases identified, the proportion of people on treatment, proportion of people who continued treatment, mortality data and viral loads). When we initially analyzed all four indicators we realized simplifying to focus on the most sensitive measure of performance, which was RIC, would allow us to characterize facility performance to guide the selection of the most widely performing facilities for the interviews.**

**_______________________________________­­­­­­­­­­­­­­­­­­­_______________________________**

Reviewer #2: PLOS-One_Review
Program Sustainability Post PEPFAR Direct Service Support in the Western Cape, South Africa
December 2020

This article provides a timely and important inquiry into the sustainability of donor-supported global health programs. This is an important topic that should have far greater attention paid to it in the global health literature. There are several aspects of the paper that require further development, including more extensive engagement with the global health literature, greater attention to the context of the Western Cape province, and relative to their proposed structure of program development, consideration of the role that community-based HIV/AIDS activists have played in South African HIV/AIDS politics and policy across the institutional levels of the state.
Thus, while the paper provides an interesting perspective on an important topic, it requires major revision before it can be considered for publication. As such, my recommendation for this paper is for it be revised and resubmitted for review.

First and foremost, there is insufficient engagement with influential approaches to program design in the field of global health. While these approaches have been primarily framed around patient-centered health outcomes rather than sustainability, since the authors focus on retention in care (RIC) as the mechanism through which sustainability is defined, the analysis is focused on similar parameters. With respect to approaches that the authors should review and engage with, Partners in Health (PiH) have advocated for an accompaniment approach to program development that involves extended engagement with across governmental sectors, civil society, and communities. In addition, Health Alliance International (HAI) has advocated for ‘diagonal’ approaches to global donor funding that involve coordination and engagement with local governmental and civil society actors and organizations to increase the impact of donor aid on communities and produce sustainable interventions.

**Thank you for these references. My understanding from the PIH accompaniment approach is that this was focused on patient adherence to ART, which does not relate to program sustainability. While these examples clearly provide assistance, improving continuity of care for HIV defaulters our research was focused on program sustainability not individual continuity of care.**

**(Mukherjee JS, Barry D, Weatherford RD, Desai IK, Farmer PE. Community-Based ART Programs: Sustaining Adherence and Follow-up. *Curr HIV/AIDS Rep*. 2016;13(6):359-366. doi:10.1007/s11904-016-0335-7)**

**Yes, HAI has advocated for SWAp and various other approaches to coordinating programs and asking for broader health system support, but vertical programing budgets have outweighed the Ministry of Health’s attempts at changing these practices. I have added a section on this the discussion.**

These are but two of many approaches to sustainability for global health programs that the authors would do well to consider as part of their analysis. The analysis is also quite shallow relative to the history of global/international health, which is a bit troubling. In short, the authors are not contextualizing their findings relative to important developments/histories in the field, which limits the impact and significance of their findings.

**Thank you for this comment. I added a few paragraphs in the Program Sustainability section.**

In addition, there is very little attention paid to the contextualization of this case study. I find the research to be valuable, but there is very little attention to precisely how and why the Western Cape stands out relative to other regions in South Africa relative to HIV/AIDS treatment.

**I added a paragraph about how the Western Cape is different from the rest of South Africa. Please refer to lines 209-216.**

I have commented on this at length below and will refer the authors to my input there, but this point also links to their recommendations for program design, particularly their focus on facility managers. Taking individual leadership as a key factor without contextualizing the conditions within which facility managers operate diminishes the potential contribution. My own experience with facility managers in the Western Cape was that those who work closely with the community and HIV/AIDS activists also found success, but these sort of ties and shared governance did not find their place in the analysis or the program design recommendations the authors provide.

**I regret that I did not formally enquire about the ties between activism and closeness with the community. I found some facility managers were skeptical of HIV activists and others were HIV activists themselves.**

Regarding the parameters for the analysis, I appreciate that the authors have made the decision to publish their quantitative data separately. However, to exclude the financial data on sustainability from this paper entirely is quite limiting. This sort of data would provide the necessary context for understanding why some clinics were able to undertake the transition better than others, which remains unclear in the paper. It would also strengthen the comparative value of the paper, as we don’t know the size of Pepfar programs relative to public health budgets in the Western Cape, which would seem critical to contextualizing why the province was able to absorb former Pepfar-funded NGO staff members into the public health system. It may be the case that other societies that are Pepfar recipients would be able to undertake similar measures, but we don’t have the necessary information to undertake these sort of comparative exercises.

**I completely agree with you. In my original proposal we included the financial data for individual NGO’s and different health facility levels but when we started collecting information it was impossible and would have totally changed the thesis and for that reason left out. It remains a valuable suggestion. I hope someone who has an accounting background would take this up. It is surprising it has not been conducted by PEPFAR. It would be invaluable. We thought we could do it but the focus was not on financial sustainability but on what makes for sustainable outcomes.**

While it is likely beyond the scope of this paper, I would encourage the authors to think more critically about the broader power dynamics within which their research is situated. In thinking about “transitions” away from global donor funding, it is not only Pepfar that is noteworthy, but also the Global Fund. In short, this is a very important topic that they are engaging with, the implications of which are very much a life and death matter for many around the world, even more so as the COVID-19 pandemic brings with it economic contraction.

Since many recipient countries remain caught in a situation where conditionalities associated with debt repayment mean that they cannot increase health spending without commensurate increases in GDP, the looming crisis of “transitions” in global health funding mean that many societies will be facing declining levels of donor support along with shrinking health budgets. While a drug-resistant HIV epidemic is one possible entailment of this, generalized increases in mortality, under-nutrition, and suffering are also likely.

All of which is to say, I know that I have been quite critical with my review, but I have done so because I see the potential impact of this line of research and would like for the paper to reach its full potential. As such, please take my comments and critique in the constructive spirit in which they have been given, as I believe that this is an important topic that needs far greater attention.


Comments
Line 62
Retention in Care (RIC) – define acronym with first use

**Noted and edited. ______________________________________________________________________**

Line 105 – 108
It is true that many countries began to augment transnational donor funding during this time, but it is also important to note that the 2008 financial crisis produced a levelling-off in donor funding, and that with access to HIV/AIDS treatment and small decreases in HIV incidence, that the number of PLHIV continued to increase during this time, necessitating that recipient governments augment their HIV/AIDS programs.

**Noted and edited.**

**______________________________________________________________________**

Line 108-109
It would also be important to note that the World Bank recategorized country income levels according to new criteria during this time, which led to restrictions on donor flows, such as with the delineation of middle-income countries (MICs). That these criteria are based on aggregate income levels and do not take into account levels of social inequality has been an important critique of this process.

**Noted and edited.**

**___________________________________________________________________**

Line 140
A bit more background on why the donor community defines sustainability along financial lines is a very important issue to contextualize. From the roots of early international health programs led by the Pan-American Health Organization (PAHO), which were funded by the Rockefeller Foundation, to the rise of selective primary healthcare and cost-recovery amid structural adjustment in the 1980s, there is a clear thread whereby donors and countries that are able to exert authority within international institutions express power by defining program sustainability and impact according to criteria that they set, one of which is financial sustainability.

**Thank you for this comment. I added a section which provides more context.**
**______________________________________________________________________**

Line 150 – 152
Please see James Pfeiffer’s research on Mozambique on how Pepfar-funded interventions interact with public health systems, as it is more complex than these programs “working inside” public health clinics, day hospitals, etc.

**Thank you for this comment. I added a paragraph which explains PEPFAR’s influence on the health system.
_______________________________________­­­­­­­­­­­­­­­­­­­_______________________________**

Line 163-164
This is a good point, but it is also important to note that the emphasis on access to treatment was also based on the logic of treatment as prevention (TasP), or that people on HIV/AIDS treatment with undetectable viral loads could not transmit the virus to other people.

**This concept came later. While this was the rationale for the TB programs there was limited evidence until much later and used in the PEPFAR program.**

**______________________________­­­­­­­­­­­­­­­­­­­_______________________________**

Line 169
On the lack of formal analysis of the transition in Pepfar programmatic leadership: who was to funded this? Who should have dedicated staff to examine this? There is an implicit critique here but it is left undefined, leading the reader to assume that the South African government should have done so. Is this the authors’ position? If so, please define.

**This is a good point that is addressed in the Conclusion section**.

______________________________________________________________________
Line 174
A bit more context here on why the Western Cape was distinct is important. The concentration of tertiary services and expertise is significant, but that is informed by the uneven historical development of health services around white, urban populations that began during the colonial era, continued during apartheid, and has not been resolved during the post-apartheid era. Also, HIV/AIDS programs were developed earlier in the province under the guidance of Fareed Abdullah and his team, particularly with PMTCT, which national government intervened to stop during the dark days of the Mbeki era. But support from the Global Fund in the province, which came earlier than the rest of the country, set up the Western Cape as an early success story and a province that has continued to exhibit stronger relative program management than others. Also, I believe that a similar process had already been undertaken with a Global Fund grant in the late 2000s, so there would have been institutional knowledge on how to manage the transition of donor programs from the government to an NGO.

**In your last sentence I assume you mean transition from NGO to government?
I consulted with the WCDoH, who said there was never a formal transfer of Global Fund human resources or programming over to government. Global Funded human resources applied for WCDoH posts when they were advertised.**
______________________________________________________________________

Line 194
I hope that the financial aspects of the transition are not left out entirely, as that would weaken the robustness of the analysis considerably.

**We** **analyzed PEPFAR expenditure figures for South Africa from 2007-2015.**
**While this allowed us to see the dip in funding from 2012-2014 in 2015 PEPFAR funding increased again when the Global AIDS Coordinator decided to fund direct service again.**

|  | SAG | PEPFAR |
| --- | --- | --- |
| 2007/08 | 6000000000 | 2700000000 |
| 2008/09 | 8000000000 | 4600000000 |
| 2009/10 | 9800000000 | 4050000000 |
| 2011/12 | 13300000000 | 3870000000 |
| 2012/13 | 14900000000 | 3900000000 |
| 2013/14 | 17800000000 | 3690000000 |
| 2014/15 | 17289000000 | 4200000000 |
| 2015/16 | 19395000000 | 4730000000 |

**Figure 9: PEPFAR and SAG Expenditure HIV and TB**

**2007-2015 (ZAR)***

**Source**:

2007-2010: South African Consolidated HIV and TB Spending Assessment 2007/8-2009/10

2011-2014: South African HIV and TB Investment Case, Reference Report

2014-2016: Consolidated HIV and TB Spending Assessment 2014/15-2016/17

**_______________________________________­­­­­­­­­­­­­­­­­­­_______________________________**

Line 200
In terms of defining RIC, a bit more would be helpful. I’m assuming that you are referring to people living with HIV/AIDS (PLHIV) who were adhering to treatment that were lost to follow up (LTFU)/did not adhere after the transition on Pepfar programs? Clarifying this would be helpful.

**The definition of RIC used in this study is also used by the WCDoH. RIC was calculated per health facility per year among adults (age >15),**

**First line + Second line + Third Line + Clients stopped ART / (Total on treatment – Total transferred out)**

**“Total on treatment” includes the HIV clients who transferred into the health facility, via a formal or silent transfer. Silent transfers were considered new ART initiates, in the absence of a patient tracking system. Mortality dropped out of the RIC calculation. A decision was made based on a sensitivity analysis that an alternative RIC definition would not significantly change the outcome.**

**_______________________________________­­­­­­­­­­­­­­­­­­­_______________________________**

Line 216
Define primary healthcare (PHC) for first use

**I edited PHC. Thank you.**

**_______________________________________­­­­­­­­­­­­­­­­­­­_______________________________**

Line 232
Of what level were the staff nurses? Clinic Nurse Practitioners would be the assumption, but please define that for the reader.

**The level of nurse was 1 staff nurse and 1 clinical nurse practitioner. I noted these distinctions in the paper. See lines 287-289.**

**_______________________________________­­­­­­­­­­­­­­­­­­­_______________________________**

Line 253
Please define modified grounded theory

**This was also a misunderstanding on my part. It should just be grounded theory.**

**_______________________________________­­­­­­­­­­­­­­­­­­­_______________________________**

Line 274
Defining the donor as an NGO is confusing here, since Pepfar is a donor program funded by the US gov’t. If you are referring to a primary recipient organization (PRO) that is working with secondary recipient organization (SRO), then that needs to be defined. Also, Pepfar, the Global Fund, and several other major global health programs are public-private partnerships (PPPs), so thinking through how you define these relationships is important, as simply labeling the donor and NGO limits the applicability and impact of your findings. Also, is the grantee always local government? This section needs to be thought through much more.

**Thank you for this comment. I edited my paper referring to either “local government, NGO or donor (ie.PEPFAR).**

**­­­­­­­­­­­­­­­­­­_______________________________________­­­­­­­­­­­­­­­­­­­_______________________________**

Line 277
Were there any parameters for defining what the coordinating position would be enabled to do/oversee? This seems a bit general and undefined since it is the key point in the section.

**This person would be responsible for ensuring the transparency of donor funded activities and work with government to ensure the program is integrated into the local health system. I clarified this in the paper.
_______________________________________­­­­­­­­­­­­­­­­­­­_______________________________**

Line 280
Donor funded organization? Seems like there is a missing word here

**This has been edited.
_______________________________________­­­­­­­­­­­­­­­­­­­_______________________________**

Line 286 - 288
This is an important point that you are making, but it is not sufficiently contextualized. What you are observing is that the responsibility for a successful transition in skill transfer is decentralized and falls to the level of a facility manager. It would lead one to assume that the better capacitated facilities would therefore be better suited to have successful transfers. Since the areas with highest HIV prevalence and greatest need for skills transfer (the peri-urban townships) also tend to have over-burdened heal facilities, this is a critical point that should be further contextualized.

**To clarify, my point is that the skills transfer needs to both centralized at a provincial level and decentralized at a facility level. The provincial level is needed so that essential donor funded staff are transferred to the public health system, which needs coordination in terms of adequate budgeting. At a health facility level, Health Facility Managers should ensure that donor funded staff who are being let go train local health facility staff in their job responsibilities.**

**I clarified this point in the paper.**

**_______________________________________­­­­­­­­­­­­­­­­­­­_______________________________**

Line 303
Define NIMART for the reader

**Edited. Thank you**

**_______________________________________­­­­­­­­­­­­­­­­­­­_______________________________**

Line 314 – 31
Again, this had already been done by the WCDoH previously, with the transition of clinic management for the HIV/TB clinics established by Médecins sans Frontières (MSF) in Khayelitsha. There were some bumps in the road with these transitions, and it was a learning process. So, again, the success here is not surprising but the result of previous experience in navigating precisely this sort of transition.

**My understanding is the MSF work in Khayelitsha Site B for the past 20 years. They have worked in the same health facility. I know that they have conducted research on transitioning patients into adult ART treatment programs. I am aware of their work on adherence clubs, which was later adapted and scaled up throughout the province.**

**The WCDoH did not have experience transitioning programs and human resources as vast as PEPFAR. PEPFAR was very different in that they also were not transparent with their work so the WCDoH was not aware of the vast amount of programming in the province. PEPFAR was supporting 435 human resources in the Western Cape in 2012. So yes, the WCDoH may have had some experience transitioning some patients into the health system, but it was not at the scale of the PEPFAR experience.**

**_______________________________________­­­­­­­­­­­­­­­­­­­_______________________________**

Line 331
Again, the fact that the extra labor associated with ensuring a sustainable transition is being transferred to the facility level is really an important point. It is not being managed by the donor, provincial health, or city health, but by the facility manager. There would be a huge range of outcomes to be expected then, which would depend not only on the personal attributes of the facility manager, but the extent to which the facility is fully staffed, operational, etc. so that the manager has the ability to focus on the transition. Also, I would assume that CNPs would play a critical role in facilitating this transition, as they often have the clearest understanding of staffing needs, shortages, and areas where increased efficiencies can be achieved. Their labor, however, is often rendered invisible in this process, which is problematic.

**These are all very good points and are highlighted in the Conclusion section.**

**_____________________­­­­­­­­­­­­­­­­­­­_______________________________­­­­­_________________**

Line 352
Define IMCI

**Noted and edited.
_______________________________________­­­­­­­­­­­­­­­­­­­_______________________________**

Line 391
It is helpful that you are addressing the contextual factors here, but this is insufficient to frame your findings, which are quite particular to the Western Cape.

 **Point taken this will be noted in the conclusion ___________________________________­­­­­­­­­­­­­­­­­­­_______________________________**

Line 396
It would be helpful to mention the role of HIV/AIDS activism in producing this change, as this was critical to enabling the shifts you identify.

**Good point added to sentence about political support.
_______________________________________­­­­­­­­­­­­­­­­­­­_______________________________**
Line 400
There is a long-standing debate on the limits of vertical, disease-specific interventions and their lack of sustainability. Proponents of using “vertical” interventions to strengthen the broader health system (horizontal) interventions have advocated for doing precisely what you advise here, to create “diagonal” programs that use vertical funding streams for health systems strengthening. Health Alliance International (HAI) has done work that has modeled this approach in Mozambique is one of the most significant examples of the potential impact and success of this approach in the global health. In general, I would recommend linking your case study and discussion of findings to the global health literature, as your case study is constructed as a stand-alone example, when in reality it is part of a broader conversation on how best to channel donor funding to improve health outcomes.

**As the reviewer states this is a long running debate and will be addressed again in the conclusion**

_____________________________________________________________________

Line 411
Is it because facilities are smaller or that they may be struggling to meet the level of need in communities with high burdens of disease?

**The reason for this is usually smaller health facilities with few staff are not able to let health staff just focus on one disease. So the donor funded HIV staff will usually get absorbed into the facilities and end up being a generalist, attending to all patients. This point was added to the conclusion.**

**_______________________________________­­­­­­­­­­­­­­­­­­­_______________________________**

Line 422
Does it make sense to mention the 90-90-90 goals for the first time in the conclusion? If this is the aim of the paper, then it would make sense to introduce this goal (which we are projected to miss significantly by the way) earlier in the paper.

**I added a section at the beginning (line 260-264) regarding the 90-90-90 goals.**

**____________________________________­­­­­­­­­­­­­­­­­­­_______________________________**

Line 437
Who are national stakeholders? Does they include civil society and PLHIV or HIV/AIDS activists?
Also, shouldn’t provincial government work with facilities and communities to understand local needs?

**I defined these points in the paper.**

**_______________________________________­­­­­­­­­­­­­­­­­­­_______________________________**

Line 452
What are local champions? Who defines needs? What is the role of the community in this process?

**I defined these points in the paper in Table 4 .**

**_____________________________­­­­­­­­­­­­­­­­­­­_______________________________**

Line 461
It might be useful to include the provincial treasury in the key stakeholders meetings, since presumably they will need to plan several years in advance if a transition will create greater budgetary demands for the health sector. The medium term expenditure framework (MTEF) requires such advance planning for budgetary processes.

**I defined these points in the paper (see Table 5).**
**_______________________________________­­­­­­­­­­­­­­­­­­­_______________________________**

Line 465
I would include the community or some proxy thereof in the final box in this section. The role of HIV/AIDS activists as counsellors and mentors who were also consulted by the WCDoH early on in the development of HIV/AIDS programs was vital to their success.

**Thank you. I added this detail to the paper.**
**_______________________________________­­­­­­­­­­­­­­­­­­­_______________________________**

Line 471
Who is funding the staffing/capacity required to develop the transition plan? Is this being done by external consultants?

**Thank you for this comment. I clarified this point in the paper**.

**_______________________________________­­­­­­­­­­­­­­­­­­­_______________________________**
Line 472
The recommendation to have the skills transfer managed at the provincial level is contradicted by your evidence, which showed that facility managers oversaw this process.

**This is not what I was conveying. Please see my comment from lines** 286 – 288 **above**

Line 474
Given that the entire focus of your paper is to emphasize the importance and lack of research on how care is affected by a donor transition, I am very surprised that there is not a post-transition phase for research or monitoring/evaluation. Your proposed flow of program transition would therefore reproduce the precise issue that your paper aims to rectify.

**This is a very good point. I have added a section that includes the post-transition time period (see Table 8).**

6. PLOS authors have the option to publish the peer review history of their article ([what does this mean?](https://journals.plos.org/plosone/s/editorial-and-peer-review-process#loc-peer-review-history)). If published, this will include your full peer review and any attached files.


If you choose “no”, your identity will remain anonymous but your review may still be made public.


**Do you want your identity to be public for this peer review?** For information about this choice, including consent withdrawal, please see our [Privacy Policy](https://www.plos.org/privacy-policy).

Reviewer #1: No

Reviewer #2: **Yes:** Theodore Powers
