# Supplementary material for: Program sustainability post PEPFAR direct service support in the Western Cape, South Africa
Source: PLoS One. 2021 May 24;16(5):e0251230. doi: 10.1371/journal.pone.0251230 (PMC8189145; doi:10.1371/journal.pone.0251230)
Supplement: Attachment — Submitted filename: Response to Reviewers #2.docx [file pone.0251230.s004.docx]

**Responses to Reviewers #2**

Dear Plos One,

Please find my responses in Bold to the reviewers.

Thank you.

Jessica Chiliza

Journal Requirements:

Please review your reference list to ensure that it is complete and correct. If you have cited papers that have been retracted, please include the rationale for doing so in the manuscript text, or remove these references and replace them with relevant current references. Any changes to the reference list should be mentioned in the rebuttal letter that accompanies your revised manuscript. If you need to cite a retracted article, indicate the article’s retracted status in the References list and also include a citation and full reference for the retraction notice.

**Reference #39 was added to include the decrease in global HIV funding as 90-90-90 targets were implemented.**

Additional Editor Comments (if provided):

 Reviewers' comments:

Reviewer's Responses to Questions

**Comments to the Author**

1. If the authors have adequately addressed your comments raised in a previous round of review and you feel that this manuscript is now acceptable for publication, you may indicate that here to bypass the “Comments to the Author” section, enter your conflict of interest statement in the “Confidential to Editor” section, and submit your "Accept" recommendation.

Reviewer #1: (No Response)

Reviewer #2: All comments have been addressed

2. Is the manuscript technically sound, and do the data support the conclusions?

The manuscript must describe a technically sound piece of scientific research with data that supports the conclusions. Experiments must have been conducted rigorously, with appropriate controls, replication, and sample sizes. The conclusions must be drawn appropriately based on the data presented.

Reviewer #1: No

Reviewer #2: Yes

3. Has the statistical analysis been performed appropriately and rigorously?

Reviewer #1: N/A

Reviewer #2: N/A

4. Have the authors made all data underlying the findings in their manuscript fully available?

The [PLOS Data policy](http://www.plosone.org/static/policies.action#sharing) requires authors to make all data underlying the findings described in their manuscript fully available without restriction, with rare exception (please refer to the Data Availability Statement in the manuscript PDF file). The data should be provided as part of the manuscript or its supporting information, or deposited to a public repository. For example, in addition to summary statistics, the data points behind means, medians and variance measures should be available. If there are restrictions on publicly sharing data—e.g. participant privacy or use of data from a third party—those must be specified.

Reviewer #1: Yes

Reviewer #2: Yes

5. Is the manuscript presented in an intelligible fashion and written in standard English?

PLOS ONE does not copyedit accepted manuscripts, so the language in submitted articles must be clear, correct, and unambiguous. Any typographical or grammatical errors should be corrected at revision, so please note any specific errors here.

Reviewer #1: Yes

Reviewer #2: Yes

6. Review Comments to the Author

Please use the space provided to explain your answers to the questions above. You may also include additional comments for the author, including concerns about dual publication, research ethics, or publication ethics. (Please upload your review as an attachment if it exceeds 20,000 characters)

Reviewer #1: N/A

Reviewer #2: The authors have significantly strengthened the paper through revision, and my recommendation is that the article be accepted for publication pending minor revisions, which are detailed below.

Line 75
This study suggests

**Noted and edited.**

Line 101
In 2008, or following 2008?

**Noted and edited.**

Line 111
Missing word after “increased”

**Noted and edited.**

Line 119
PEPFAR misspelled. Also, “after PEPFAR withdrawal” or “after the withdrawal of PEPFAR funding”

**Noted and edited.**

Line 122
Insert “and reductions in” prior to “tracing systems”

**Noted and edited.**

Line 171
It looks like there is an incomplete sentence here with “The donor community capacity”

**Noted. It looks part of my sentence was missing. Thank you.**

Paragraph starting on Line 197
This is an excellent addition to the argument.

**Thank you.**

Section starting on Line 203
This is very helpful for situating the particularity of the Western Cape.
**Thank you.**

Paragraph starting on Line 253
It might be useful to signal that the 90-90-90 rhetoric emanating from the UN has coincided with the leveling off of donor funding and the “transition” processes initiated by PEPFAR and the Global Fund. In short, while we have the tools to “end HIV/AIDS”, funding that would have otherwise supported this approach has been withdrawn, which has undermined this program. Perhaps this would fit best in the conclusion, but it would be worth mentioning, as this critical dynamic seems to be lost on many.

**Thank you. I added a sentence on line 253 and in the conclusion with regard to this decrease in funding.**

Line 350
Consider rewording to: “understand the context, and local policy, and have…”
**Noted and edited.**

Line 351
Consider rewording to: “to the context, which builds trust and results in more”

**Thank you. I edited the whole sentence (lines 350 and 351) to the following:**

**“When an NGO has an established office in the geographic region, they understand the context, local policy and have strong relationships with government , which builds trust and results in more sustainable outcomes.”**

Line 356
Consider rewording to: “and at a decentralized level.”

**Noted and edited.**

Line 450
Consider rewording to: “centralized level: either the provincial or district level.”

**Noted and edited.**

Line 455
Consider rewording to: “important for government and the NGO to be”

**Noted and edited.**

Line 467
This interview excerpt has already been used (Line 419). Please delete one of these so that there is not repetition.

**Oh my! Thank you very much for picking this up. Noted and Edited.**

Line 491
Consider rewording to: “the main concern is that if the public-sector workforce and infrastructure are undermined.”

**Noted and edited.**

Line 511
Start new paragraph with: “Donors”

**Noted and edited.**

Line 546
Consider rewording to: “need to be intentionally”

**Noted and edited.**

Line 554
Under “Grantee”: “Ideally have an Establish a donor coordination”

**Edited. Thank you!**

Line 577
Perhaps consider including a wider array of inputs on the transition planning process. Certainly, while the donors will appreciate the objectivity of an external consultant, this is a critical moment in ensuring the long-term sustainability of the program, and there should therefore be a clear and thorough consultation process that involves the full range of stakeholders.

**Thank you for this comment. I have added a few additional inputs.**
